# Factor Structure of the 10-Item Perceived Stress Scale and Measurement Invariance Across Genders Among Chinese Adolescents

**DOI:** 10.3389/fpsyg.2020.00537

**Published:** 2020-04-09

**Authors:** Xiqin Liu, Yajun Zhao, Jingguang Li, Jing Dai, Xiuli Wang, Song Wang

**Affiliations:** ^1^The Clinical Hospital of Chengdu Brain Science Institute, MOE Key Lab for Neuroinformation, University of Electronic Science and Technology of China, Chengdu, China; ^2^School of Sociology and Psychology, Southwest Minzu University, Chengdu, China; ^3^College of Teacher Education, Dali University, Dali, China; ^4^Huaxi MR Research Center, Department of Radiology, West China Hospital of Sichuan University, Chengdu, China

**Keywords:** Perceived Stress Scale, confirmatory factor analysis, measurement invariance, Chinese adolescents, stress

## Abstract

**Background:**

Chinese adolescents encounter a lot of stressors, such as academic burden and parental pressure. However, little is known about their perception of stress. The 10-item Perceived Stress Scale (PSS-10) is a widely used instrument to measure individuals’ appraisal of global stress in academic research and clinical practice. The current study aimed to evaluate the best-fit factor structure model of the PSS-10 and the measurement invariance across genders in Chinese adolescents.

**Methods:**

A total of 1,574 Chinese senior high school students completed the PSS-10 (mean age = 15.26 ± 0.56 years, female = 54%). Confirmatory factor analysis (CFA) was conducted to determine the factor structure of the PSS-10. Multigroup CFA was carried out to test the measurement invariance of the PSS-10 across genders. A subsample (*N* = 1,060) answered additional questionnaires measuring stressful life events, anxiety, and depression to examine the convergent and concurrent validity of the PSS-10.

**Results:**

The two-factor model was supported [i.e., χ^2^ (34) = 332.224, *p* < 0.001; non-normal fit index (NNFI) = 0.901, comparative fit index (CFI) = 0.925, root mean square error of approximation (RMSEA) = 0.075, standardized root mean square residual (SRMR) = 0.051]. Importantly, the model exhibited strong measurement invariance across female and male groups. Furthermore, the PSS-10 had adequate convergent validity for stressful life events (number: *r* = 0.13, *p* < 0.001; impact: *r* = 0.23, *p* < 0.001) and could explain incremental variance in predicting anxiety (Δ*R*^2^ = 0.13, β = 0.38, *p* < 0.001) and depression (Δ*R*^2^ = 0.16, β = 0.41, *p* < 0.001), suggesting excellent concurrent validity.

**Conclusion:**

A two-factor model best fits the structure of PSS-10 among Chinese adolescents, with strong measurement invariance between gender groups, demonstrating its validity for assessing perceived stress among Chinese adolescents.

## Introduction

It has been argued that adolescence is a transition period in one’s life in which one undergoes biological, social, and psychological changes (McNamara, 2000; Tsai et al., 2015a, b, 2018; Lin and Tsai, 2016; Strong et al., 2016; Lee et al., 2017; Foulkes and Blakemore, 2018; Blakemore, 2019). Many psychological disorders, such as anxiety and depression, begin at adolescence and persist into adulthood (Blakemore, 2019). One of the most important reasons for the increased vulnerability to psychological health problems during this period is that adolescents encounter increasingly more stressors (i.e., academic stress, peer pressure, and parental pressure) (Kouzma and Kennedy, 2002, 2004; Deb et al., 2015; Seiffge-Krenke, 2019), and many adolescents may not have well-developed coping strategies because of limited life experiences (Moeini et al., 2008). When there is an imbalance between environmental demands and adolescents’ coping capacity, people will perceive stress (Cohen et al., 1997), which may be linked not only to unhealthy behaviors such as cigarette smoking and alcohol use (Wills and Shiffman, 1985; Cohen and Lichtenstein, 1990) but also to psychological health problems, including anxiety and depression (Lupien et al., 2009). Specifically, it has been suggested that adolescents under East Asian culture (e.g., China) are more likely to show higher depression and lower life satisfaction than the European counterparts due to the emphasis on academic performance (Stankov, 2013). The academic stress among Chinese adolescents comes from a heavy workload and fierce competition for university entrance (Cai et al., 2010) and parents’ high expectations on their academic achievement (Woo et al., 2004; Chan et al., 2017), which undermines their psychological health (Tsai et al., 2013). Hence, it is essential to evaluate Chinese adolescents’ perceived stress to be able to relate this measure to their health. Given a lack of research about their subjective perception of stress, the present study sought to evaluate the 10-item version of the Perceived Stress Scale (PSS-10; Cohen, 1988), one of the most widely used measure of global perceived stress, in a large sample of Chinese senior high school students (*N* > 1,000), in order to confirm whether this scale could be utilized in Chinese adolescents.

The PSS was originally developed as a 14-item scale (PSS-14) that assesses how respondents found their lives unpredictable, uncontrollable, and overloaded, which was designed for use in community samples with at least a junior high education (Cohen et al., 1983). Since the PSS items focus on the general nature of feelings and thoughts about stress rather than specific events or experiences, this scale is suggested to measure the global level of stress in any population (Cohen et al., 1983, 1994). Specifically, many studies have utilized the PSS to measure self-reported stress in various adolescent samples such as middle school students (e.g., Yosipovitch et al., 2007; Edwards et al., 2014) and clinical adolescents (e.g., Martin et al., 1995; Siqueira et al., 2000). While the PSS-14 shows adequate validity and reliability, the shorter 10-item version (PSS-10) was reported to show superior psychometric properties and has been recommended for use in future research (Cohen, 1988; for a review, see Lee, 2012). The PSS-10 was derived from the 10 items with high factor loadings from the original PSS-14 based on a sample of 2,387 US residents (Cohen, 1988). Since its development, the PSS-10 has been translated into over 25 languages and has been validated all over the world (Cohen, 2014). The PSS-10 possesses adequate internal consistency with Cronbach’s alpha coefficients ranging from 0.67 to 0.91 (e.g., Roberti et al., 2006; Siqueira Reis et al., 2010; Ng, 2013; Taylor, 2015; Denovan et al., 2019; Kaya et al., 2019), moderate convergent validity with stressful life events (e.g., Mitchell et al., 2008), and good concurrent validity with mental health problems such as depression and anxiety (e.g., Örücü and Demir, 2009; Perera et al., 2017; Baik et al., 2019). In particular, the PSS has been translated in Chinese and shown acceptable reliability and validity in adult samples such as postnatally distressed women (Chen et al., 2000), urban residents (Yang and Huang, 2003), working adults (Chu and Kao, 2005), police women (Wang et al., 2011), cardiac patients (Leung et al., 2010), elderly service workers (Ng, 2013), and university students (Lu et al., 2017). However, validations of the PSS-10 only focused on adult samples in previous studies, and no study has evaluated the PSS-10 in adolescents. To the best of our knowledge, the present study is the first to validate the PSS-10 in Chinese adolescents.

The factorial structure of the PSS-10 remains controversial. Although studies have consistently identified a two-factor structure of the PSS-10 with factor distinction corresponding to item directionality (i.e., negatively phrased items versus positively phrased items) (e.g., Cohen, 1988; Golden-Kreutz et al., 2004; Roberti et al., 2006; Siqueira Reis et al., 2010; Wang et al., 2011; Barbosa-Leiker et al., 2013; Taylor, 2015; Lu et al., 2017; Baik et al., 2019), researchers hold different opinions about the understanding of the distinction between the two factors. Cohen (1988) originally posited that the distinction only related to statement directionality (negative versus positive) and was irrelevant, thus the PSS-10 assessed a single construct. However, other researchers have argued that the distinction reflects separate components of the stress experience, thus measuring two factors. Specifically, negatively phrased items were labeled as “Perceived Helplessness” or “Perceived Distress,” and positively phrased items were labeled as “Perceived Self-Efficacy” or “Perceived Coping” (Golden-Kreutz et al., 2004; Roberti et al., 2006; Barbosa-Leiker et al., 2013). Another important issue concerns whether the factor structure underlying the PSS-10 is equivalent across gender groups. Previous studies have indicated that women scored higher on both the PSS-10 total scores and subscale scores than men (Leung et al., 2010; Andreou et al., 2011; Lesage et al., 2012; Barbosa-Leiker et al., 2013). However, some believe that these gender differences are due to measurement bias, that is, the idea that women are more inclined to score higher on negatively phrased items (Gitchel et al., 2011), instead of true gender differences (Lavoie and Douglas, 2012). To rule out this possibility, an increasing number of studies have tested the measurement invariance of the PSS-10 across genders and have found that the scale does not exhibit measurement bias against males and females (e.g., Barbosa-Leiker et al., 2013; Taylor, 2015; Reis et al., 2017; Denovan et al., 2019). However, whether this measurement invariance in the PSS-10 holds for Chinese adolescent is still unknown. Therefore, the goal of this study was to evaluate the factor structure of the PSS-10 and examine whether the measurement structure is equivalent across gender groups in Chinese adolescent students.

To address these questions, we first carried out confirmatory factor analysis (CFA) to test the factor structure of the PSS-10. Second, to examine whether the PSS-10 measures the same latent structure in females and males, we conducted a multigroup CFA to test measurement invariance (i.e., configural, metric, and scalar invariance) across gender groups. In addition, we validated its internal consistency reliability and validity in the current sample. Specifically, the convergent validity of the PSS-10 was measured for stressful life events [Adolescent Self-Rating Life Events Checklist (ASLEC)] because perceived stress may increase as the result of cumulative stressful life events, and the impact of such events may reflect stressor appraisal measured by the PSS-10 (Cohen et al., 1983). Additionally, concurrent validity was examined by testing whether PSS-10 scores could predict anxiety [Screen for Child Anxiety Related Emotional Disorders (SCARED)] and depression [Depression Self-Rating Scale for Children (DSRSC)] beyond stressful life events, age, and gender, given that the perception of stress may be an early symptom of anxiety and depression (Cohen et al., 1983).

## Materials and Methods

### Participants and Procedure

A total of 1,614 Chinese senior high school students in 10th grade participated in this study. Forty participants (2.48%) were excluded from analyses because values for the PSS-10 were missing completely at random [χ^2^ (112) = 103.323, *p* = 0.709] (Little, 1988; Schlomer et al., 2010; Kang, 2013). Thus, 1,574 participants were included in the analyses (856 females, 718 males; age = 13–17 years, mean age = 15.26 ± 0.56 years). The average age for the male group (mean age = 15.34 ± 0.59 years) was higher than that for the female group (mean age = 15.20 ± 0.52 years) [*t*_(1,572)_ = 5.02, *p* < 0.001]. These participants were part of a larger longitudinal project investigating the behavioral and neural mechanisms underlying adolescents’ mental health, academic achievement, and social cognition in Chengdu, China (Wang et al., 2017, 2018; Li et al., 2018a, b). All participants were from three general local public high schools and were recruited *via* a class presentation to introduce the project by researchers from West China Hospital of Sichuan University and Chengdu Education Bureau. Because the participants completed a battery of surveys across several waves in the project, only part of the data including PSS measurement was included in the present study. In addition to the PSS, several other questionnaires were administered to measure stressful life events (ASLEC), anxiety (SCARED), and depression (DSRSC). Of all 1,574 participants, 1,060 participants (601 females, 459 males; mean age = 15.26 ± 0.54 years) completed all the measures. All tests were written in simplified Chinese in a pencil-and-paper format, and each participant completed the tests in his/her classroom. To make the participants feel confident about the confidentiality of their responses, two research assistants supervised the test sessions, and teachers were not present. The current study was approved by the local research ethics committee of the West China Hospital of Sichuan University. Written informed consent was obtained from all participants and their guardians prior to testing.

### Measures

#### PSS-10

The PSS-10 is a self-reported scale to measure the global level of perceived stress (Cohen, 1988). This scale includes two factors (Golden-Kreutz et al., 2004; Roberti et al., 2006; Örücü and Demir, 2009; Barbosa-Leiker et al., 2013): Factor 1 (Perceived Helplessness) is made of negatively phrased items (i.e., items 1, 2, 3, 6, 9, and 10; e.g., “In the last month, how often have you felt nervous and stressed”); and Factor 2 (Perceived Self-Efficacy) is made of positively phrased items (i.e., items 4, 5, 7, and 8; e.g., “In the last month, how often have you felt that things were going your way”). The items of the PSS-10 used in this study were selected from the Chinese version of the PSS-14 that was translated by Chu and Kao (2005) and has shown high internal consistency and associations with negative mental health such as anxiety and depression in Chinese adults (Chu and Kao, 2005; Chu, 2010). Furthermore, words and expressions were slightly modified to suit our sample. Particularly, to ensure the accuracy and readability of each item, five 10th-grade students were first asked to answer the revised scale and then interviewed to indicate the readability and suitability of each item. The interviews were conducted by a group including the first author, the last author, and a class teacher. The group had a discussion after the interviews and reached a consensus that each item is readable and suitable for high school students. Participants were required to rate how often they felt a certain way over the past month on a five-point Likert scale (1 = never, 2 = rarely, 3 = sometimes, 4 = often, and 5 = always). Scores for positively phrased items were reversed to obtain the total score and subscale score for Factor 2 (Perceived Self-Efficacy). The total score of the PSS-10 ranged from 10 to 50, and a higher score indicated a higher level of perceived stress. Previous studies have suggested that the PSS-10 shows adequate internal consistency, test–retest reliability, and validity across different populations (for a review, see Lee, 2012).

#### Adolescent Self-Rating Life Events Checklist

To evaluate the convergent validity of the PSS-10, we incorporated the ASLEC to measure stressful life events experienced over the past 12 months (Liu et al., 1997). This measure includes 27 stressful life events from multiple domains: family pressure, academic pressure, interpersonal conflicts, physical health problems, and others. Participants were first instructed to answer “yes” or “no” to the question asking whether the given event had ever happened to them. Then, they were asked to rate the impact of each experienced event on a five-point Likert-type scale ranging from 1 (not at all) to 5 (extremely severe). The number of stressful life events for each participant was obtained by counting all experienced events. The impact of stressful life events was obtained by summing scores for all events. Of note, the impact of unexperienced events was coded as 1 (not at all). The ASLEC has been reported to have satisfactory validity and reliability in previous studies (Liu et al., 1997) and has been widely used to assess the number and impact of stressful life events in Chinese adolescents (e.g., Liu et al., 2000; Liu and Tein, 2005; Zheng et al., 2012). In the current study, the Cronbach’s alpha coefficient for the ASLEC was 0.92, indicating excellent internal reliability.

#### Screen for Child Anxiety Related Emotional Disorders

To examine the concurrent validity of the PSS, we employed the SCARED (Birmaher et al., 1997; Muris et al., 1998), which is a self-reported instrument measuring symptoms of Diagnostic and Statistical Manual of Mental Disorders, Fourth Edition (DSM-IV)-linked anxiety disorders in children 9–18 years old. The SCARED contains 41 items across five factors: somatic/panic, general anxiety, separation anxiety, social phobia, and school phobia (Birmaher et al., 1999). Participants were instructed to rate how frequently they have experienced each symptom in the last 3 months using a three-point scale, in which 0 represents “never,” 1 represents “sometimes,” and 2 represents “often.” The SCARED total score and subscale scores were computed by summing up the corresponding items, and a higher score indicated a higher level of anxiety. The Chinese version of the SCARED has proven to have good psychometric properties (Wang et al., 2002). In the current study, the overall Cronbach’s alpha coefficient of the SCARED was 0.94. For the five factors, the coefficients were 0.89, 0.84, 0.74, 0.84, and 0.68, respectively, indicating adequate internal reliability.

#### Depression Self-Rating Scale for Children

To examine the role of the PSS score as a predictor of depression, the DSRSC (Birleson et al., 1987) was used. This unidimensional instrument has 18 items and covers the major areas of mood disturbance. Participants were asked to rate how frequently they had experienced certain feelings in the previous week, ranging from 0 (never) to 2 (often). The total score of the DSRSC was obtained by summing the ratings of all items, with a higher total score indicating a higher level of depression. The Chinese version of the DSRSC has demonstrated satisfactory reliability and validity and is applicable to assess depressive symptoms in Chinese children and adolescents (Su et al., 2003; Yin et al., 2012). The Cronbach’s alpha coefficient for the DSRSC in this study was 0.81, indicating satisfactory internal reliability.

### Data Analysis

First, descriptive statistics of each PSS-10 item [i.e., mean, standard deviation (SD), skewness, kurtosis, and discrimination index (DI)] were calculated by SPSS (IBM, SPSS version 20, 2011). The absolute skewness and kurtosis values smaller than 1 indicate a normal distribution of the scores of items (Marcoulides and Hershberger, 2014). The DI is a measure of how well an item is able to distinguish between respondents with high scores and low scores. This measure is generally computed by subtracting the mean score of participants in the lower group (27%) from the mean score of those in the upper group (27%) and dividing the result by the maximum possible discrimination score (Kelley et al., 2002). DI values of 0.40 and above are regarded as being very good items, and those of 0.20 or less are regarded as being poor items (Ebel, 1954; Hopkins et al., 1990; Hu and Xie, 2012).

Second, we carried out a CFA to test whether the two-factor model can be replicated in the current sample using Amos 22.0 with maximum likelihood estimation method. We used multiple indices to evaluate the model’s goodness of fit as recommended by Kline (2005) and Byrne (2001): non-normal fit index (NNFI), comparative fit index (CFI), root mean square error of approximation (RMSEA), and standardized root mean square residual (SRMR). An acceptable model fit is indicated by an NNFI of >0.90, a CFI of > 0.90, an RMSEA of < 0.08, and an SRMR of <0.08 (Hu and Bentler, 1999; Byrne, 2001; Kline, 2005). Notably, to further determine the superiority of the two-factor model, we also evaluated a one-factor model and compared it with the two-factor model by using a chi-squared difference test (Duckworth and Quinn, 2009; Li et al., 2018b).

Third, measurement invariance concerns whether items and structural factors mean the same thing to different groups (Cheung and Rensvold, 2002). To evaluate measurement invariance across genders, we tested the configural, metric, and scalar invariance using a multigroup CFA in Amos 22.0. These tests are hierarchically nested by increasing levels of cross-group equality constraints (Gregorich, 2006; Kuhn and Holling, 2009). Configural invariance tested whether the factor structure of the PSS-10 is the same across gender groups. Metric invariance tested whether the intercorrelations of the PSS-10 items were equivalent across groups by constraining the factor loadings to be equivalent. Scalar invariance tested if the group mean differences were due to differences in the construct by fixing the intercepts to be equal across groups. Having change values of CFI and RMSEA (ΔCFI and ΔRMSEA) smaller than 0.01 is recommended as an appropriate criterion indicating measurement invariance (Cheung and Rensvold, 2002; Chen, 2007).

Additionally, we calculated the Cronbach’s alpha coefficients to evaluate the internal reliability of the PSS-10. We then evaluated the convergent validity of the PSS-10 by examining Pearson correlation coefficients between the total and subscale scores of the PSS-10 and the ASLEC. Finally, the concurrent validity of the PSS-10 was verified by testing its role in predicting anxiety (SCARED) and depression (DSRSC) based on hierarchical regression analyses. We only used the total score of the PSS-10 to index perceived stress in regression analyses to simplify analysis, given that the two subscale scores showed similar results. These analyses were conducted with SPSS (IBM, SPSS Version 20, 2011).

## Results

### Descriptive Statistics

Table 1 presents the descriptive statistics for each item. As shown, the mean values ranged from 2.59 to 3.10, and the SD values ranged from 0.78 to 0.93. The scores of all items were normally distributed (skewness: −0.32∼0.51; kurtosis: −0.44∼0.52). In addition, all PSS-10 items had good discrimination, with DI values ranging from 0.46 to 0.57.

**TABLE 1:** Descriptive statistics of the PSS-10.

Items	Means	SD	Skewness	Kurtosis	DI
1. Been upset	2.98	0.89	0.12	0.14	0.55
2. Unable to control	2.92	0.89	0.16	0.06	0.54
3. Nervous and stressed	3.10	0.93	0.32	–0.23	0.57
4. Felt confident	2.82	0.92	–0.12	–0.44	0.56
5. Going your way	3.09	0.80	–0.15	0.22	0.48
6. Could not cope	2.94	0.78	0.29	0.52	0.46
7. Control irritations	2.99	0.78	–0.26	0.14	0.48
8. On top of things	3.22	0.88	–0.32	–0.01	0.50
9. Been angered	2.59	0.91	0.51	0.11	0.49
10. Could not overcome	2.82	0.93	0.26	–0.03	0.54

*N = 1,574. DI, discrimination index; PSS-10, 10-item Perceived Stress Scale; SD, standard deviation.*

### Confirmatory Factor Analysis

**FIGURE 1:**
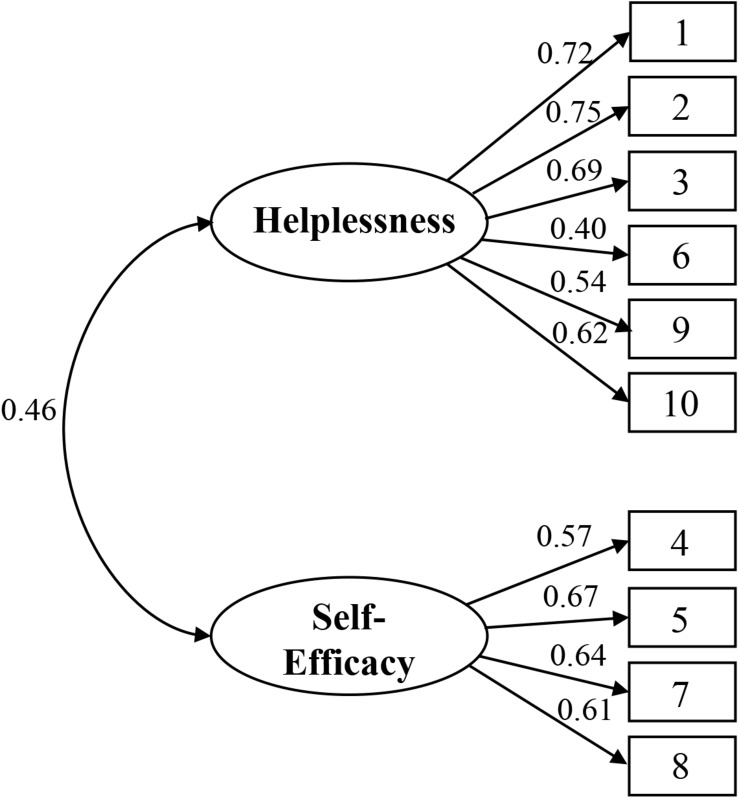
Standardized factor loadings for the two-factor model of the 10-item Perceived Stress Scale (PSS-10) (*N* = 1,574).

Confirmatory factor analysis was used to determine the factor structure of the PSS-10. All fit indices suggested a good data fit for the two-factor model [i.e., χ^2^ (34) = 332.224, *p* < 0.001; NNFI = 0.901, CFI = 0.925, RMSEA = 0.075, SRMR = 0.051] but not for the one-factor model [i.e., χ^2^ (35) = 1,056.412, *p* < 0.001; NNFI = 0.670, CFI = 0.743, RMSEA = 0.136, SRMR = 0.101]. By comparing these two models, we found that the two-factor model demonstrated a better fit than the one-factor model, as indicated by a significant chi-squared difference [Δχ^2^ (1) = 724.118, *p* < 0.001]. The factor loadings ranged from 0.40 to 0.75 for negatively phrased items (Factor 1: Perceived Helplessness) and from 0.57 to 0.67 for positively phrased items (Factor 2: Perceived Self-Efficacy), and all loadings were significant at the 0.001 level (Figure 1). In summary, CFA supported the two-factor model of the PSS-10.

### Measurement Invariance Across Gender Groups

Measurement invariance tests were subsequently conducted using a multigroup CFA to determine whether the PSS-10 can represent the two-factor structure across gender groups.

First, configural invariance was performed by analyzing the two gender groups without constraining equality across the groups. The result showed that the configural invariance model (M1) fitted the data very well (CFI = 0.919, NNFI = 0.904, RMSEA = 0.050) (Table 2), and all factor loadings were significant (*p* < 0.005). These results indicated that the two-factor structural patterns are similar across gender groups.

**TABLE 2:** Model fit indices for measurement invariance across genders.

Model	Δχ^2^	df	CFI	NNFI	RMSEA	Comparison	Δχ^2^	ΔCFI	ΔRMSEA
M1	398.48	76	0.919	0.904	0.050				
M2	411.90	79	0.916	0.904	0.052	M2 vs. M1	13.42^∗∗^	0.003	0.002
M3	454.94	89	0.908	0.907	0.051	M3 vs. M2	43.04^∗∗∗^	0.008	0.001

*N = 1,574. CFI, comparative fit index; NNFI, non-normal fit index; RMSEA, root mean square error of approximation; M1, configural model; M2, metric model; M3, scalar model. ^∗∗^p < 0.01, ^∗∗∗^p < 0.001.*

Next, a metric invariance model (M2) was tested by constraining the factor loadings to be equal across groups. The results showed a good fit (CFI = 0.916, NNFI = 0.904, RMSEA = 0.052) (Table 2). Although Δχ^2^ (*p* < 0.01) was significant when compared to M1, the change value of CFI (ΔCFI = 0.003) and an RMSEA (ΔRMSEA = 0.002) smaller than 0.01 provide evidence to support the metric invariances.

Third, we conducted a scalar invariance test by restricting the item intercepts to be invariant across gender groups. As shown in Table 2, the model fit indices of the scalar invariance model (M3) are adequate (CFI = 0.908, NNFI = 0.907, RMSEA = 0.051). Compared to M2, although Δχ^2^ (*p* < 0.001) was significant, no significant changes occurred in the CFI (ΔCFI = 0.008) or RMSEA (ΔRMSEA = 0.001) based on the cutoff, which was 0.01 for ΔCFI and ΔRMSEA, suggesting that the means of the two constructs were equivalent across gender groups.

Taken together, the results of measurement invariance indicated that the structural factors of the PSS-10 were equivalent across gender groups.

### Reliability

The Cronbach’s alpha coefficients for the PSS-10 and the two subscales (Perceived Helplessness and Perceived Self-Efficacy) were 0.79, 0.80, and 0.71, respectively.

### Convergent and Concurrent Validity

We examined the relationship between the PSS-10 and the ASLEC and found that the total PSS-10 score was significantly related to both the number (*r* = 0.13, *p* < 0.001) and the impact (*r* = 0.23, *p* < 0.001) of the ASLEC. We also tested the correlations between the two subscales of the PSS-10 and the ASLEC, and the results showed that Factor 1 (Perceived Helplessness) was significantly related to both the number (*r* = 0.12, *p* < 0.001) and the impact (*r* = 0.21, *p* < 0.001) of the ASLEC, whereas Factor 2 (Perceived Self-Efficacy) was only associated with the impact (*r* = 0.11, *p* < 0.001) but not the number (*r* = 0.05, *p* = 0.10) of the ASLEC. Importantly, for the total and subscale scores of the PSS-10, the associations with the impact of the ASLEC were all significantly higher than those with the number of the ASLEC (total score: Steiger’s *Z* = 4.89, *p* < 0.001; Factor 1: Steiger’s *Z* = 4.38, *p* < 0.001; Factor 2: Steiger’s *Z* = 2.89, *p* < 0.01). These results suggest that perceived stress was more highly correlated with conceptually similar constructs (i.e., subjective feeling of the impact of stressful life events) than with objective concepts (i.e., the number of stressful life events), indicating good convergent validity.

To verify the concurrent validity of the PSS-10, correlation analyses were first conducted to test the relationship between perceived stress and anxiety and depression. Scores of the PSS-10 and its two subscales were correlated with scores of the SCARED and its five subscales, as well as the DSRSC (Table 3). Next, hierarchical regression analyses were conducted to examine the incremental validity of perceived stress on anxiety and depression, after variance related to other variables has been explained. The results revealed that perceived stress explained an additional 13% of the variance when predicting anxiety (Δ*R*^2^ = 0.13, β = 0.38, *p* < 0.001) and an additional 16% of the variance when predicting depression (Δ*R*^2^ = 0.16, β = 0.41, *p* < 0.001) beyond the variance already explained by age, gender, ASLEC number, and ASLEC impact (Table 4). Altogether, the PSS-10 has satisfactory concurrent validity in predicting anxiety and depression.

**TABLE 3:** Pearson correlation coefficients between the PSS-10 and other measures.

Scale	PSS-10	Helplessness	Self-efficacy
ASLEC			
Number	0.13^∗∗∗^	0.12^∗∗∗^	0.05
Impact	0.23^∗∗∗^	0.21^∗∗∗^	0.10^∗∗^
SCARED	0.43^∗∗∗^	0.41^∗∗∗^	0.23^∗∗∗^
Somatic/Panic	0.37^∗∗∗^	0.36^∗∗∗^	0.17^∗∗∗^
General Anxiety	0.43^∗∗∗^	0.42^∗∗∗^	0.22^∗∗∗^
Separation Anxiety	0.29^∗∗∗^	0.29^∗∗∗^	0.16^∗∗∗^
Social Phobia	0.33^∗∗∗^	0.25^∗∗∗^	0.24^∗∗∗^
School Phobia	0.31^∗∗∗^	0.28^∗∗∗^	0.13^∗∗∗^
DSRSC	0.42^∗∗∗^	0.38^∗∗∗^	0.29^∗∗∗^

*N = 1,060. ASLEC, adolescent self-rating life events checklist; DSRSC, depression Self-Rating Scale for children; PSS-10, 10-item perceived stress scale; SCARED, the screen for child anxiety related emotional disorders. ^∗∗^p < 0.01, ^∗∗∗^p < 0.001.*

**TABLE 4:** Hierarchical regression models for predicting anxiety and depression.

	Anxiety (SCARED)			Depression (DSRSC)		
	*B*	β	Δ*R*^2^	*B*	β	Δ*R*^2^
Step 1			0.10^∗∗∗^			0.04^∗∗∗^
Age	–0.09	0.00		0.43	0.04	
Gender	3.23	0.11^∗∗∗^		–0.15	–0.01	
ASLEC Number	–0.11	–0.05		0	0	
ASLEC Impact	0.29	0.33^∗∗∗^		0.06	0.18^∗∗∗^	
Step 2			0.13^∗∗∗^			0.16^∗∗∗^
Age	0.01	0.00		0.47	0.05	
Gender	2.70	0.09^∗∗^		–0.37	–0.03	
ASLEC Number	–0.02	–0.01		0.04	0.05	
ASLEC Impact	0.19	0.21^∗∗∗^		0.02	0.06	
PSS-10	1.06	0.38^∗∗∗^		0.44	0.41^∗∗∗^	

*N = 1,060. ASLEC, adolescent self-rating life events checklist; DSRSC, depression self-rating scale for children; PSS-10, 10-item perceived stress scale; SCARED, the screen for child anxiety related emotional disorders. ^∗∗^p < 0.01, ^∗∗∗^p < 0.001.*

## Discussion

In this study, we evaluated the factor structure, measurement invariance, reliability, and validity of the PSS-10 in a large sample of Chinese senior high school students. CFA supported a two-factor model of the PSS-10. Multigroup CFA further indicated that the two-factor structure of the PSS-10 was upheld across genders. Additionally, the PSS-10 had acceptable internal consistency, satisfactory convergent validity for stressful life events, and good concurrent validity in predicting anxiety and depression. To our knowledge, this is the first study to evaluate the PSS-10 in Chinese adolescents and to demonstrate measurement invariance across genders. Overall, our results suggested that the PSS-10 could be applied to Chinese adolescents to measure perceived stress.

The two-factor structure found in the current sample was in line with findings of previous studies on the use of the PSS-10 in other populations, such as university students (e.g., Roberti et al., 2006; Örücü and Demir, 2009; Lu et al., 2017), general adult samples (e.g., Siqueira Reis et al., 2010; Wang et al., 2011; Ng, 2013; Taylor, 2015; Baik et al., 2019), and clinical patients (e.g., Golden-Kreutz et al., 2004; Leung et al., 2010). Most researchers have argued that the distinction between the two factors may represent separate components of the stress experience (Baik et al., 2019). The first factor has been interpreted as “Perceived Helplessness” or “Perceived Distress” (negatively phrased items), which reflects a lack of control and negative emotions; the second factor has been interpreted as “Perceived Self-Efficacy” or “Perceived Coping” (positively phrased items), which taps positive feelings and perceptions of confidence (e.g., Golden-Kreutz et al., 2004; Roberti et al., 2006; Barbosa-Leiker et al., 2013). Nevertheless, given that Cohen (1988) originally posited that the distinction between the two factors was irrelevant and only reflected item directionality, and the two-factor may arise from the problem of method biases (Schmitt, 1994), we suggest that the subscale scores should be cautiously used and interpreted in future studies.

Based on the configural, metric, and scalar invariance tests, we found that the two-factor model of the PSS-10 was equivalent for male and female adolescents, suggesting that the PSS-10 scores may not be affected by gender bias. This is similar to the results of previous studies with English versions (e.g., Barbosa-Leiker et al., 2013; Taylor, 2015; Denovan et al., 2019) and German versions (e.g., Reis et al., 2017) of the PSS-10. The present study, to the best of our knowledge, is the first to establish measurement invariance in the Chinese PSS-10 across genders, which provides support for using the PSS-10 to assess gender differences in Chinese adolescents.

Further analyses supported the internal consistency, reliability, and validity of the PSS-10 in assessing Chinese adolescents. The internal consistency of the present study is comparable to that from previous studies in Chinese populations (e.g., Cronbach’s alpha: 0.67–0.87; Wang et al., 2011; Ng, 2013; Lu et al., 2017), indicating adequate internal reliability. Convergent validity was tested for stressful life events. Cohen et al. (1983) originally investigated the relationship between the PSS-14 and stressful life events and found that there was a small to moderate correlation between the number and impact of stressful life events and the PSS-14 in three American college student samples. Previous studies have also reported good convergent validity of the PSS-10 with other conceptually similar measures, such as the Life Event Scale (Mitchell et al., 2008). Consistently, the present study revealed that PSS-10 scores were significantly related to both the number and the impact of the ASLEC. Further regression analyses supported the concurrent validity of the PSS-10 for assessing anxiety and depression. Previous studies demonstrated correlations between the PSS-10 scores and anxiety/depression in Chinese university students (Lu et al., 2017) and policewomen (Wang et al., 2011). In addition to the correlational results, our study uses hierarchical regression analyses to further reveal the incremental validity of the PSS-10 beyond predictors of stressful life events, suggesting the distinctness of perceived stress beyond other theoretical related constructs that may be predictive of anxiety and depression.

The present study had two limitations that should be considered. First, the sample in this study only covered senior high school students, with a relatively narrow age range. Although this sample achieved sufficient statistical power to observe existing effects (Mackey et al., 2015), further investigations are needed to validate our findings in other adolescent students (e.g., junior high school students). Second, we only tested gender invariance. Future studies should test measurement invariance across other groups, such as across age groups or cultural groups. Despite these limitations, the present study adds to the literature by demonstrating that the PSS-10 is a sound measure of perceived stress among Chinese senior high school students with a stable two-factor structure across female and male groups, and with acceptable reliability and validity. Future research and clinical practice could use this scale to measure perceived stress in this population and to compare gender differences in the scores.

## Conclusion

Our findings provide evidence to use the PSS-10 in future studies to investigate the perceived stress in Chinese adolescents and to compare the gender differences. Future directions in research may consider the cross-cultural measurement invariance in adolescents. Moreover, our findings support the use of the PSS-10 in clinical settings to determine the severity of the load of the stressful events among Chinese adolescents and guide effective interventions to decrease their stress symptoms, especially those related to the educational system.

## Data Availability Statement

The datasets generated for this study are available on request to the corresponding author.

## Ethics Statement

The studies involving human participants were reviewed and approved by the local research ethics committee of the West China Hospital of Sichuan University. Written informed consent to participate in this study was provided by the participants’ legal guardian/next of kin.

## Author Contributions

SW and JL conceived the research. SW and YZ performed the research. XL, JD, and XW analyzed the data. XL wrote the manuscript.

## Conflict of Interest

The authors declare that the research was conducted in the absence of any commercial or financial relationships that could be construed as a potential conflict of interest.
